# Complete androgen insensitivity syndrome in a 13-year-old Lebanese child, reared as female, with bilateral inguinal hernia: a case report

**DOI:** 10.1186/s13256-021-02738-0

**Published:** 2021-04-17

**Authors:** Stephanie Farah, Dana El Masri, Kamal Hirbli

**Affiliations:** Department of Endocrinology, Diabetes and Metabolism. Lebanese, American University Medical Center, LAUMCRH, Beirut, Lebanon

**Keywords:** Androgen insensitivity syndrome, Amenorrhea, Inguinal hernia, Case report

## Abstract

**Background:**

Androgen insensitivity syndrome is a rare X-linked disorder of sex development, caused by mutations in the androgen receptor. In this case, a 13-year-old child, reared as female, presenting for primary amenorrhea, was diagnosed with complete androgen insensitivity syndrome.

**Case presentation:**

A 13-year-old Caucasian child, reared as female, presents with primary amenorrhea. Physical examination revealed female appearance and a short vagina with blind-ended pouch. Laboratory examination showed high levels of testosterone and anti-Müllerian hormone; uterus and ovaries were absent. Karyotype confirmed a 46,XY pattern. Deoxyribonucleic acid analysis of the androgen receptor gene revealed a homozygous mutation p.R856C in exon 7. Gender was assigned as female, and she was started on hormonal therapy and underwent gonadectomy.

**Conclusion:**

Androgen insensitivity syndrome comprises a large spectrum of presentations. High index of suspicion is needed. Investigation of girls with bilateral inguinal hernia is critical.

## Background

Androgen insensitivity syndrome (AIS) is a rare X-linked disorder of sex development, occurs in 1 out of 20,000 births, and is caused by mutations in the androgen receptor (AR) gene. This, in turn, makes the genetically XY male resistant to the actions of androgen hormones, resulting in a spectrum of changes, according to the severity of androgen resistance, ranging from partial to full female phenotype [1]. A few retrospective studies have estimated the incidence of complete androgen insensitivity syndrome (CAIS) to be 0.8% to 2.4% in girls with inguinal hernias [2]. In Lebanon, only two case reports of coexistence of Kallmann syndrome and complete androgen insensitivity syndrome were reported in two sisters from Lebanese consanguineous parents [3]. Given its rarity and underreporting, as well as the importance of early diagnosis and treatment, we share our case of CAIS in a 13-year-old patient, reared as female, with a history of inguinal hernia and presenting with primary amenorrhea.

## Case presentation

A 13-year-old Caucasian patient, reared as female, presented initially to our clinic for primary amenorrhea evaluation in May 2019. Clinical examination revealed normal intellectual function and feminine habitus (weight 50 kg, height 171 cm) and voice, with development of both breasts and pubic hair growth at Tanner stage 3 (the bud is enlarged beyond the areola, with no separation of the contours of the areola from the breast; in pelvic area, hair is dark and rough). Gynecological examination revealed well-developed labia, small clitoris, and a short vagina (4 cm) with blind-ended pouch; thus, the cervix could not be visualized on speculum examination. Medical history included bilateral inguinal hernias, surgically repaired at the age of 2 years. Family history is otherwise irrelevant. The patient is an only child, whose parents are divorced. She is a top student at school and a professional dancer.

Previous exams revealed normal gonadotropin levels with follicle-stimulating hormone (FSH) 1 mUI/mL, luteinizing hormone (LH) 20 mUI/mL, and estradiol 29 pmol/L. Nevertheless, both total testosterone (32 ng/mL) and anti-Müllerian hormone (AMH) 212.9 μg/L (normal 1.52–9.95 μg/L) levels were elevated. Further workup revealed the absence of uterus and ovaries, hypoplastic vagina, and intraabdominal gonads, likely testes, seen by pelvic magnetic resonance imaging (MRI). The karyotype was mapped, confirming a 46,XY pattern. DNA analysis for sequencing the entire coding sequence of the AR gene was completed in August 2019 and revealed the presence of a homozygous mutation p.R856C (c.2566C>T) in exon 7 of the AR gene, consistent with CAIS.

The patient, reared as female, was referred to our obstetrics and gynecology department to undergo laparoscopic removal of the undescended testes to avoid the risk of malignancy.

**Fig. 1.**
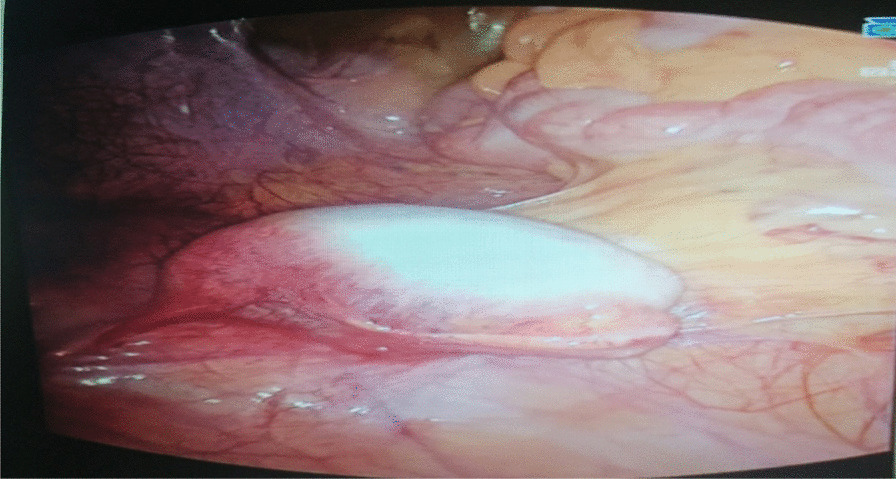
Surgical specimen

**Fig. 2.**
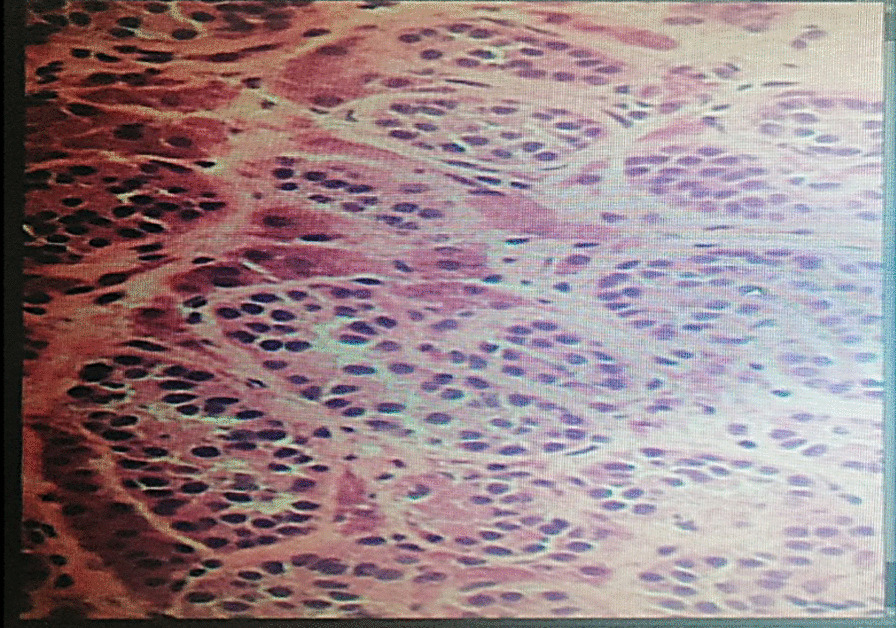
Histopathology of the specimen: both gonads showing testicular tissue with embryonal-type seminiferous tubules and Leydig cells

The testes were found inside the abdominal cavity. They were subsequently dissected and removed (Fig. 1). Histopathology revealed two testes with atrophic seminiferous tubules containing only Sertoli cells, associated with Leydig cell hyperplasia (Fig. 2). No signs of malignancy were identified. Genital reconstruction was not done because the patient had normal-appearing female external genitals, and she was started on estradiol oral replacement therapy to prevent the regression of secondary sexual characteristics and the consequences of estrogen deficiency. The patient returned 1 year postsurgery. Her breasts had clearly increased in size, and she had gained 5 kg. She is following a healthy diet and still exercises regularly. She is very intelligent and brave at school, though she is becoming more aggressive and impulsive and is losing interest in things that she used to care about, such as her relationships with friends. She continued to take her hormones on a regular basis. She was reluctant to receive any psychological or social support. Otherwise, she faced no financial or language-related challenges.

## Discussion and conclusions

AIS is an X-linked disorder of sex development caused by mutations in the AR gene located on chromosome Xq I1.12. Over 1000 mutations, as well as some larger structural alterations, have been identified in patients with all forms of AIS, and these mutations can be spread out over the whole coding region [4]. Spontaneous mutations can also give rise to disease without any family history.

Genetically XY males with these mutations do not virilize in the typical pattern, despite the presence of bilateral testes and serum testosterone concentrations within or above the usual male reference range [5]. They are typically resistant to the actions of androgen hormones. This translates to an inability of target tissue to recognize and use testosterone, resulting in a spectrum of changes, according to the severity of androgen resistance, ranging between partial and full female phenotype [1, 6]. These clinical phenotypes can be classified into three categories: complete, partial, and mild forms [7].

In either case, affected individuals have normal testes with normal production of testosterone and normal conversion to dihydrotestosterone. Because the testes produce normal amounts of Müllerian-inhibiting factor, affected individuals do not have fallopian tubes, a uterus, or a proximal vagina [1, 8]. Thus, the testes provide the natural levels of estrogens through the aromatization of testosterone.

CAIS is a rare condition with an estimated incidence of between 1:20,000 and 1:99,000 in genetic males [9]. This condition is consistently overlooked at birth, and frequently diagnosed at puberty, when the patient reports primary amenorrhea such as in our case. Nevertheless, earlier childhood identification could prevent morbidity and improve quality of life for these subjects.

Although inguinal hernias are uncommon in female infants, they are a well-known presentation of CAIS. The possibility that female infants presenting with inguinal hernia might have CAIS has been the subject of several studies. In a large series of 124 infants and children with inguinal hernias, 3 were found to have CAIS [10]. A similar incidence was reported in other comparable series of patients [11, 12]. A few retrospective studies have estimated the incidence of CAIS to be 0.8% to 2.4% in girls with inguinal hernias [2, 9]. On the other hand, around 50–80 % of patients with CAIS have a history of inguinal hernias [13, 14].

In light of these findings, the development of inguinal hernia in phenotypic female infants should raise suspicion of CAIS [2, 15]. Some clinicians recommend karyotyping or biopsy of a gonad within the hernial sac [16], while others recommend measuring vaginal length [17] to screen for CAIS in prepubertal girls with inguinal hernia.

Whole-exome sequencing is one of the most valuable tools for the detection of Mendelian diseases, including CAIS. The AR gene is located on the X chromosome (Xq I1.12) and contains eight exons [18]. To date, more than 500 mutations in AR have been described, including point mutations, frameshift mutations leading to premature termination of transcription, and gross deletions, as well as small deletions or insertions scattered around the entire sequence of the gene [19]. The p.R856C mutation found in our case was confirmed to affect the binding of the androgen receptor and ligand [20, 21]. This mutation was first reported in 2006 [22]. Accordingly, appropriate genetic counseling is advisable to relatives at risk.

Girls with CAIS have a normal pubertal growth spurt and feminize at the time of expected puberty secondary to aromatization of androgen to estrogen, and testicular tumors do not usually develop until after this time [23]. However, carcinoma *in situ* of the testis has been described in prepubertal girls [24]. Gonadectomy is then indicated after puberty to avoid the increased risk. Then, hormone replacement is initiated after, or at the time of, expected puberty if gonadectomy was performed prepubertally [25].

Corrective surgery of the urogenital tract for affected patients who are phenotypic females or are raised as female with vaginal dilator therapy or vaginoplasty prior to the time when an active sexual life is contemplated is desirable.

Finally, it is essential to provide the patient and family with psychological support and appropriate education about the condition as soon as the diagnosis is made, as suggested by current practice guidelines [26, 27]. Such disclosure prevents the unfortunate consequences of a patient learning of the diagnosis inadvertently.

We report a homozygous mutation p.R856C (c.2566C>T) in exon 7 of the AR gene in a case of complete androgen insensitivity with history of inguinal hernia during infancy. This identified mutation was different from those reported in the two Lebanese sisters (g.104222C>G and c.1617-3C>G) who were diagnosed with CAIS and Kallmann syndrome [3]. As early diagnosis of CAIS is important, investigation of girls with inguinal hernia is mandatory. Management should then focus on counseling of families and providing assistance with gender assignment, addressing the time of gonadectomy to prevent the increased tumor formation in cryptorchid testes, administering hormone replacement at the appropriate time, performing surgery as appropriate for individuals raised as male or female, and providing psychological support for the patient and family.

Complete androgen insensitivity syndrome comprises a large spectrum of presentations. High index of suspicion is needed for early diagnosis and treatment. It should be included in the differential diagnosis of primary amenorrhea, especially if the patient has history of bilateral inguinal hernia. Genetic testing is important for confirmation. Family counseling and gender assignment are highly recommended.

## Data Availability

The authors declare that data supporting the findings of this study are available within the article.
